# Sixth BHD Symposium and First International Upstate Kidney Cancer Symposium: latest scientific and clinical discoveries

**DOI:** 10.18632/oncotarget.7733

**Published:** 2016-02-25

**Authors:** Gennady Bratslavsky, Mark R. Woodford, Michael Daneshvar, Mehdi Mollapour

**Affiliations:** ^1^ Department of Urology, SUNY Upstate Medical University, Syracuse, NY, USA; ^2^ Cancer Research Institute, SUNY Upstate Medical University, Syracuse, NY, USA; ^3^ Department of Biochemistry and Molecular Biology, SUNY Upstate Medical University, Syracuse, NY, USA

**Keywords:** FLCN, Birt-Hogg-Dubé syndrome, renal cell carcinoma, clear cell renal cell carcinoma

## Abstract

The Sixth BHD Symposium and First International Upstate Kidney Cancer Symposium concluded in September 2015, in Syracuse, NY, USA. The program highlighted recent findings in a variety of areas, including drug development, therapeutics and surgical management of patients with BHD and multi-focal renal tumors, as well as multidisciplinary approaches for patients with localized, locally advanced and metastatic renal cell carcinoma.

## INTRODUCTION

Worldwide nearly 338,000 people develop kidney cancer every year, and over 100,000 people die from the disease [1, 2]. Renal cell carcinoma (RCC) is the most common type of chemotherapy-resistant kidney cancer and it is distinguishable by histopathological features as well as underlying gene mutations [3]. Clear cell renal cell carcinoma (ccRCC) is the most common type of RCC and it is closely associated with mutations of the Von Hippel-Lindau (*VHL*) tumor suppressor gene that lead to stabilization of hypoxia inducible factors (HIF-1α and HIF-2α). This is critical for tumor growth and angiogenesis in both sporadic and familial forms of this disease [4, 5]. The current standard of treatment for ccRCC are agents that target the VHL–HIF pathway, specifically VEGF, PDGF, and mTOR.

In contrast to VHL, Birt-Hogg-Dubé (BHD) syndrome is a rare inherited cancer syndrome that predisposes affected individuals to develop kidney tumors (usually not clear cell type like in VHL), pulmonary cysts, and benign skin tumors (fibrofolliculomas) [6]. Germline mutations in the tumor suppressor gene *Folliculin* (*FLCN*) cause BHD syndrome [7, 8].

FLCN behaves as a haploinsufficient tumor suppressor in skin lesions, whereas a loss of function of both alleles (usually via different “second hits”) is reported in kidney tumors. These tumors vary in histology from chromophobe RCC to benign oncocytoma, and most commonly present as hybrid tumors containing features of both chromophobe RCC and oncocytomas [9-11]. FLCN is an evolutionarily conserved, nuclear and cytoplasmic phosphoprotein that interacts with Folliculin Interacting Proteins 1 and 2 (FNIP1 and FNIP2) in a phosphorylation-dependent manner, and together they form a complex with AMPK [12]. The functional outcome of this biochemical interaction and the mechanistic details of FLCN-FNIP1 and FNIP2 interaction remain unclear. Opposing data have been reported indicating that FLCN regulates mTORC1 function *in vitro* and *in vivo* [13-16]. Despite substantial progress in BHD research there are two broad and major gaps in our knowledge: 1) Why do tumors occur in BHD patients? 2) Development of strategies to treat patients with BHD syndrome.

**Figure 1:**
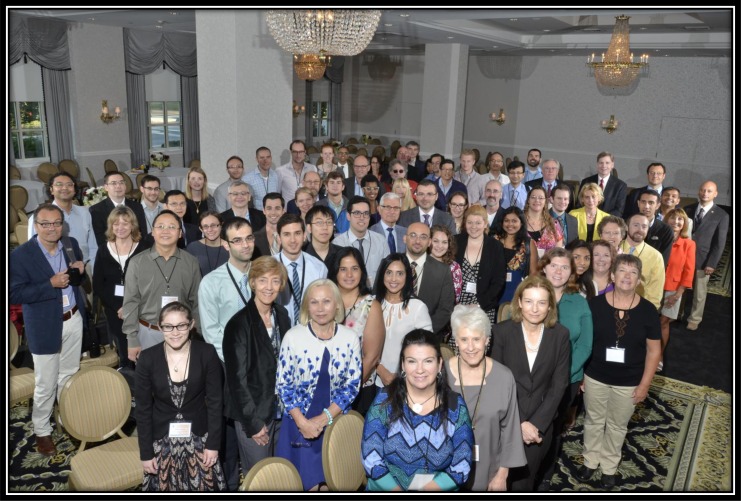
Participants of the Sixth BHD Symposium and First International Upstate Kidney Cancer Symposium

The Sixth BHD Symposium and First International Upstate Kidney Cancer Symposium organized by Gennady Bratslavsky MD (Chair, Department of Urology) and Mehdi Mollapour PhD (Head, Renal Cancer Biology Section) at SUNY Upstate Medical University, USA, brought together many of the leading researchers and surgeons from across the globe. Patients with BHD syndrome and other types of kidney cancer also attended the meeting (Figure 1).

## LATEST RESEARCH FINDINGS ON BHD AND RCC

**Figure 2:**
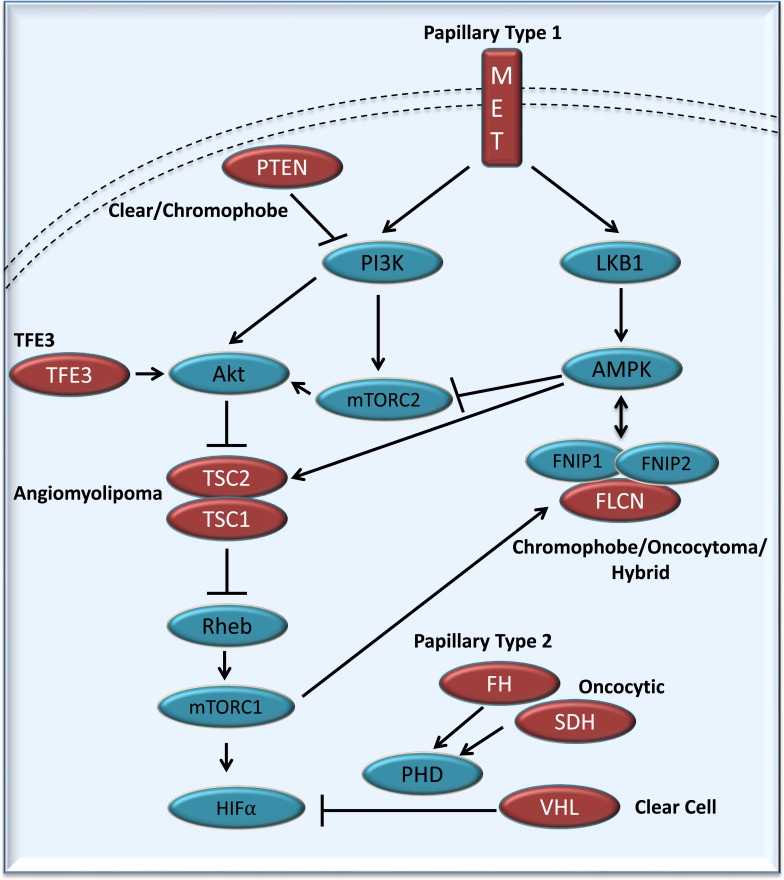
Simplified representation of the kidney cancer gene pathways Kidney cancer is fundamentally a metabolic disease with each of the kidney cancer genes (in red), *VHL*, *MET*, *FLCN*, *FH*, *SDH*, *TSC1*, *TSC2*, *PTEN* and *TFE3* disrupts the ability of cells to sense oxygen, iron, nutrients, and energy. (Figure 2 adapted from these references [23, 24]).

In the opening keynote presentation Dr. W. Marston Linehan, Chief of the Urologic Oncology Branch at the National Cancer Institute (NCI), National Institute Health (NIH), reviewed the genetic basis of kidney cancer and the importance of understanding the underlying biology for development of treatments. The rest of the first day focused on the ongoing BHD and RCC scientific research (Figure 2). Dr. Elizabeth Henske (Brigham and Women's Hospital, Harvard Medical School, Boston, USA) presented data on the pathogenic and therapeutic links between Tuberous Sclerosis Complex (TSC) and BHD. TSC is a rare multi-system genetic disease that, like BHD, causes benign tumors to grow in the kidneys, eyes, lungs, and skin. Dr. Damir Khabibullin from Henske's group presented exciting data on FLCN interacting protein armadillo-repeat containing protein plakophilin 4 (PKP4, p0071) and its role in cell adhesion and metabolism in BHD and chromophobe RCC. Dr. Maria F. Czyzyk-Krzeska (University of Cincinnati College of Medicine, Cincinnati, USA) showed that VHL and FLCN induce expression of LC3C but inhibit expression of LC3B. LC3C, but not LC3B, is specifically necessary for the autophagic destruction of midbodies. The mechanism of specificity involves the presence of a C-terminal peptide present in LC3C but not LC3B. The number of midbodies is augmented in cancer cells and stem cells indicating the role of midbodies, and therefore programs regulating their numbers, in cellular reprogramming and tumorigenicity.

Dr. Vera P. Krymskaya (University of Pennsylvania, Philadelphia, USA) showed that FLCN regulates lung epithelial cell survival and alveolar size. Her data suggested that lung cysts in BHD result from an underlying defect in alveolar epithelial cell survival attributable to FLCN regulation of the E-cadherin-LKB1-AMPK axis. Dr. Arnim Pause (McGill University, Montréal, Canada) presented two recent exciting findings from his lab. He first presented data on FLCN and AMPK conferring resistance to hyperosmotic stress via remodeling of glycogen stores. Second, using an adipose-specific *FLCN* knockout (adipo-FLCN KO) mouse model, it was shown that FLCN repression induces metabolic reprogramming of white adipose tissue through the AMPK/PGC-1α/ERRα axis. Dr. Yu Jiang (University of Pittsburgh School of Medicine, Pittsburgh, USA) showed that FLCN is a ciliary protein that functions through primary cilia to regulate mTORC1. In response to flow stress, FLCN interacts with LKB1 and recruits the kinase to primary cilia for activation of AMPK, which causes mTORC1 down-regulation. Dr. Masaya Baba's group at Yokohama City University, Yokohama, Japan, has generated a Xp11.2 translocation RCC mouse model, in which a floxed neomycin cassette followed by a PRCC-TFE3 cDNA are inserted in the Rosa26 locus. By crossing these mice with cadherin 16-Cre transgenic mice, they were able to induce kidney specific PRCC-TFE3 expression resulting in RCC development. This model will be useful to clarify the molecular mechanisms of both Xp11.2 translocation RCC and BHD-associated RCC development. Dr. Sunil Sudarshan (The University of Alabama at Birmingham, Birmingham, USA) presented data on the elevation of the putative oncometabolite l enantiomer of 2-hydroxyglutarate in the most common subtype of kidney cancer and described a novel mechanism for the regulation of DNA 5-hydroxymethylcytosine levels. Their findings provide new insight into the metabolic basis for the epigenetic landscape of renal cancer. Dr. Haifeng Yang (Thomas Jefferson University, Pennsylvania, USA) showed exciting data on the functional analysis of the tumor suppressor *PBRM1* in ccRCC. His group has identified lysine acetylation on PBRM1 as a critical PBAF binding signal. Additionally, loss of PBRM1 is associated with worse prognosis in ccRCC patients while the losses of BRM or BRG1, the enzymatic subunits of PBAF, were associated with better prognosis.

Dr. Laura Schmidt (NCI, Bethesda, USA) presented data on the germline FLCN mutation spectrum and phenotype analysis of 226 families with BHD syndrome. Renal manifestations, most frequently hybrid oncocytic tumors, developed in half of all BHD families. Although no clear genotype-phenotype correlations were observed, no renal neoplasia was reported in 3 families harboring large gene deletions encompassing the putative exon1 promoter region. Related to this topic, Dr. Jorge Toro (NCI, Bethesda, USA) also discussed current and emerging techniques for detection of FLCN mutations. He reported 87 novel FLCN germline mutations, including 40 deletions, 15 insertions, 14 missense, 12 nonsense, 4 splice-site and 2 deletion/insertions.

Dr. Angela Pacitto from Professor Tom L. Blundell's group (University of Cambridge; Cambridge, UK) reported the crystal structure of the N-terminal region of Lst4 (yeast folliculin interacting partner (FNIP1/2) ortholog) from *Kluyveromyces lactis* to 2.14 Å resolution and showed that it contains a longin domain. Folliculin and FNIP1/2 have been proposed to be members of the DENN-family of proteins, and the longin domain is the first domain of this protein fold. This work expands our understanding of the structural organization of the FLCN/FNIP complex and its role in disease. Dr. Adam Blanden from Professor Stewart Loh's lab (SUNY Upstate Medical University, Syracuse, USA) showed that the zinc metallochaperone ZMC1 could serve as a new class of experimental cancer drugs that reactivate mutant p53 by restoring proper zinc binding to several zinc-impaired mutants.

Dr. Diana Dunn and Dr. Mehdi Mollapour (SUNY Upstate Medical University, Syracuse, USA) showed that pharmacologic inhibition of c-Abl prevents Aha1 co-chaperone interaction with Hsp90, thereby hypersensitizing renal cell carcinoma (RCC) to Hsp90 inhibitors both *in vitro* and *ex vivo* [17]. Dr. Mark Woodford and Dr. Mehdi Mollapour (SUNY Upstate Medical University, Syracuse, USA) reported the identification of FNIP1/FNIP2 as new co-chaperones of Hsp90 that decelerate the chaperone cycle, therefore facilitating FLCN interaction with Hsp90, consequently ensuring FLCN stability.

## LATEST CLINICAL DISCOVERIES

The clinical day of the meeting started with keynote speaker Dr. Robert Uzzo (Fox Chase Cancer Center, Philadelphia, USA). He presented updates on the management of RCC and emphasized the importance of risk management in RCC, separately addressing both preclinical and clinical risk. He suggested that RCC risks could be divided into four different categories: 1) Tumor risk, 2) Patient risk, 3) Physician risk and 4) Hospital risk. All four risk factors are intimately linked and intersect in the decision-making process of patient management, patients' prognoses, and outcomes of various interventions. Following the notion of risks, Dr. Ilene Sussman, Director of the VHL Alliance, (Boston, USA) reported on the Cancer in Our Genes International Patient Databank (CGIP), a patient driven online databank that was created in order to measure the impact of modifiable lifestyle factors on tumor development of new VHL lesions and other related rare diseases that have a kidney cancer component. Dr. Jo Robays (Belgian Health Care Knowledge Centre, KCE, Belgium) presented on a Belgian clinical practice guideline that was created by the collaborative efforts of the Belgian Health Care Knowledge Centre (KCE) and the Belgian College of Human Genetics and the College of Oncology based on a systematic review of BHD evidence.

Dr. Mitsuko Furuya (Yokohama City University, Yokohama, Japan) discussed the clinicopathological features of 300 Japanese individuals with BHD who were investigated from 115 families. In this cohort 28 unique gene mutations were identified in the *FLCN* gene. Mutations occurred in all exons except number 8, and most mutations occurred in the exon 11-13 “hot spots”. Overall, the incidence of RCC was 19.3% (58/300). Respiratory symptoms were the most frequent phenotype, and the possible association of lung neoplasms and liver cysts within patients with BHD is still under investigation.

Dr. Nishant Gupta (University of Cincinnati, Cincinnati, USA) discussed the use of a Markov state transition model that was constructed in order to retrospectively evaluate the cost effectiveness of high-resolution computed tomography (HRCT) screening in spontaneous pneumothorax. It is reported that BHD patients have a 50-fold higher chance of pneumothorax, with approximately 24% experiencing a pneumothorax in their lifetime, and a recurrence rate of 75%. They compared the strategy of HRCT screening in patients diagnosed with BHD who then had pleurodesis to those with no HRCT screening. Based on Medicare data and costs, they reported that it was more cost effective to perform HRCT of the thorax in patients with primary spontaneous pneumothorax for screening of BHD. Dr. Paul Johannesma (VU University Medical Center, Amsterdam, The Netherlands) described findings that BHD patients have a significantly higher recurrence rate of spontaneous pneumothorax (SP) after only conservative treatment compared to invasive treatment. Given the high SP recurrence rate in BHD patients, one proposal is to engage in invasive treatment through laparoscopic surgery (total pleurectomy and pleurodesis) with the aim to completely obliterate the pleural cavity. Dr. Irma van de Beek (Department of Pulmonary Disease, Department of Clinical Genetics, VU University Medical Center, Amsterdam, The Netherlands) described a retrospective study designed to evaluate compliance of the currently advised frequency of surveillance and outcomes of surveillance in 164 patients who were diagnosed with BHD in two Dutch specialized centers. Their data indicated that compliance with advised screening for RCC was high and that ultrasound might be a sensitive and widely available imaging modality for detecting clinically relevant renal tumors in BHD patients.

Dr. Adam Metwalli (National Cancer Institute, Bethesda, USA) presented on the surgical and functional outcomes of patients with multifocal RCC. The impact of prolonged surgical time and rhabdomyolysis on multivariable analysis did not have long-term impact on surgical outcomes or renal function. In regards to patients who underwent repeat robotic partial nephrectomy (PNx), there was a higher rate of conversion from robotic to open surgery. In this same cohort, renal function was preserved, while there was increased blood loss and a higher rate of complications [18-20]. Ultimately, PNx resulted in excellent cancer control and preservation of renal function in multifocal RCC patients. Dr. Brian Shuch (Yale Cancer Center, New Haven, USA) first discussed the prediction of the clinical behavior of renal tumors by the presence or absence of a pseudocapsule and their multifocality by the presence or absence of nodal metastases. He also demonstrated data that the percentage of preserved renal parenchyma determines functional outcomes in post-partial nephrectomy patients.

Dr. Gennady Bratslavsky (SUNY Upstate Medical University, Syracuse, USA) discussed the role of cytoreductive nephrectomy in metastatic RCC. He highlighted two awaited trials (EORTC and CARMENA) that could hopefully address this question in the era of TKIs. The EORTC trial for metastatic RCC addresses a question of the appropriate sequence in RCC treatment: sunitinib followed by nephrectomy versus nephrectomy followed by sunitinib. The CARMENA trial for metastatic RCC will compare sunitinib alone versus both nephrectomy and sunitinib. Meanwhile, surgeons can use several available prognostic models to help predict of outcomes, such as the MSKCC guidelines and the international consortium on RCC [21, 22]. Future studies will further explain the sequence of first or second line therapies and the sequences of these treatment modalities along with combination therapy, immunotherapy and vaccine based treatments. Ultimately, the role of a surgeon in the management of metastatic RCC is to avoid unnecessary options and to continue supporting clinical trials until the role of cytoreductive nephrectomy is better defined.

Dr. Jason Muhitch (Roswell Park Cancer Institute, Buffalo, USA) presented recent work on immunotherapy in RCC and the results of a clinical trial that evaluated tumor-associated antigen (TAA) expression in surgically resected patient RCC lesions from RCC patients treated with high dose radiation. He also discussed how dendritic cell immunotherapy via vaccination could help promote tumor-specific T cell responses that can be effective in a subset of RCC patients. For example, the AGS-003 dendritic cell vaccine works by injecting dendritic cells that presents antigens into patients in order to elicit T cell responses. In combination with SUTENT, the phase II trial had encouraging outcomes and the large phase III has recently completed its accrual. Dr. Namitha Chittoria (VA Medical Center, Syracuse, USA) discussed the different treatment options and clinical trials for metastatic non-clear RCC (ncRCC). The Phase II ASPEN trial is the largest trial in ncRCC with 108 patients who were either treated with sunitinib or everolimus. Patients who received sunitinib improved their overall progression free survival (PFS) while everolimus did better in patients with poorer risk. The ESPN trial and the RECORD-3 trial were also presented. The optimal treatment for ncRCC remains uncertain and there is limited evidence due to the rarity of the tumor, and thus multi-institutional trials need to be conducted.

Dr. Fang-Ming Deng (NYU School of Medicine, New York, USA) presented updates on the classification of RCC. Many new tumor entities have been discovered since the 2004 WHO classification of RCC and these will be added to the 2016 WHO classification system. The newest additions to the 2016 WHO classification can be subdivided by their distinct clinical features, syndromic and familial entities, and their therapeutic implications. The newest additions include hybrid oncocytic chromophobe tumor, tubulocystic RCC, hereditary leiomyomatosis RCC syndrome-associated RCC, SDH deficient RCC and several other types. Therefore, if a renal tumor is seen with unusual clinical and morphological features, then one should consider the possibility of classification using a new RCC subtype.

## BHD AND RCC FAMILY PROGRAM

The last day of the meeting was a patient-focused open forum session led by genetic counselors Ms Lindsay Middelton (NCI, Bethesda, USA) and Ms Bonnie Braddock (Upstate Medical University, Syracuse, USA). Due to the rarity of BHD and RCC, patients affected by these conditions often have questions and concerns that cannot be answered by their local health care providers, and they do not know anyone else who has their condition. Patients and their family members had an opportunity to interact with each other and also ask general questions from clinicians and researchers who are experts in BHD and RCC field.

A short presentation by Dr. Elizabeth Henske provided analogies to help clarify the cellular roles of FLCN. Dr. Nishant Gupta explained the physiology of SP and the rationale behind choosing different treatments. Dr. Gupta and others also helped to allay some fears for patients with an elevated risk of SP. Ilene Sussman outlined the CGIP from the patient perspective, and detailed the benefits of participation from both the patient's and researcher's perspective. Dr. Jorge Toro discussed the various types of skin conditions associated with hereditary kidney cancer, available cosmetic treatments, and ongoing clinical trials. Dr. Gennady Bratslavsky discussed treatment options available for patients with BHD and renal tumors as well as importance of timely surveillance and intervention after the largest tumor reaches 3cm.

## CONCLUDING REMARKS

Considerable advances have been made within the past two years into understanding the function of *FLCN* and its involvement in BHD syndrome.

We have seen some considerable advances in the treatment of RCC in the past decade based on research that found the causes of kidney cancer. There are approximately 15 genes, including *FLCN* and *VHL*, that can cause kidney cancer and each of those genes is involved in tumorigenesis. The exchange of information between the different research areas will substantially contribute to understanding of RCC tumor initiation and progression as well as a whole new way for diagnosis and treatment.
